# Application of ultrasound in combination with other methods in gynecological disease: artificial intelligence, surgery, and drugs

**DOI:** 10.3389/fonc.2025.1567024

**Published:** 2025-09-01

**Authors:** Shaohua Shi, Chengcheng Dai, Demin Liu, Xingjie Liu

**Affiliations:** ^1^ Department of Ultrasound, Qilu Hospital of Shandong University Dezhou Hospital, Dezhou, China; ^2^ Clinical Training Center, Qilu Hospital of Shandong University Dezhou Hospital, Dezhou, China

**Keywords:** gynecological disease, ultrasound, artificial intelligence, diagnosis, treatment

## Abstract

Gynecological diseases (GyD) are related to reproductive tissue disorders such as the cervix, vagina, fallopian tubes, and ovaries, which can affect fertility. Among these diseases, we can mention endometriosis, ovarian laziness, primary ovarian insufficiency, cancers related to these tissues, and even Asherman’s disease. Considering the impact of these diseases on the population’s youth, it is imperative to develop effective methods for diagnosing, treating, monitoring, and preventing their progression. In the past, ultrasound-based methods have been used for early diagnosis of GyD, including ovarian cancer. However, in today’s era, it is essential to enhance the features of this method to ensure that patients are screened more effectively and their treatment responses can be tracked. In recent years, the spread of artificial intelligence has led to its application in various branches of medicine. Many studies have increased their efficiency by combining ultrasound and artificial intelligence methods. Additionally, the simultaneous use of ultrasound and surgery can help improve patient recovery and the success of the procedure. Additionally, various studies have utilized the combination of ultrasound-based methods and different drugs to treat GyD. In this manuscript, we will discuss the pathology of gynecological diseases, the use of ultrasound-based methods, and their combination with other methods.

## 1 Introduction

Gynecological diseases (GyD) are of great importance due to their impact on women’s health and fertility and, ultimately, the youth of the population (1). GyD is generally categorized into three distinct groups, each with its own unique pathological pathways. These include diseases related to tumors (cervical tumors, ovarian tumors) (2)Infectious diseases (fungal infections such as vaginitis) (3), and endocrine gland-related diseases (4, 5). Also, the reason for some GyD is unknown; it can be Vulvodynia with symptoms such as pain in the vulva and itching (6). Some other diseases, such as endometriosis (7), can also occur due to a combination of different mechanisms. In endometriosis, due to retrograde menstruation, cellular remains are poured into the peritoneal cavity, and endometrial-like lesions are formed there due to the dysfunction of the immune system, which can lead to abnormal bleeding and pelvic pain (8).

To prevent the progression of these diseases, early diagnosis is critical (9). However, many diagnostic methods, such as laparoscopy, are invasive and require patient-friendly treatment. In the case of GyD, methods based on ultrasound (US) can be used as non-invasive methods (10). This method is preferentially used to diagnose benign or malignant abdominal and pelvic masses caused by the ovary, adnexa, and uterus (11). There are different types of US, which can be done in two- or three-dimensional ways. However, the results of the studies have shown that the use of three-dimensional US has a higher sensitivity than the two-dimensional type in diagnosing fibroids, cysts, adnexal torsion, endometrial thickness, and uterine congenital abnormalities (nearly 100 percent) (12).

As mentioned, ultrasound can diagnose the tumor and check its exact staging. However, the use of complementary methods such as computerized tomography, artificial intelligence, and magnetic resonance imaging (MRI) can be used for correct and differential diagnosis of endometrial cancer and other GyD (13, 14). The combination of these methods is crucial for doctors to make decisions. Considering that the US can play an important role in classifying GyD stages, using it with artificial intelligence can help choose between surgery and drug treatment (15). This review will first discuss the basics of using the US to diagnose GyD types and then how to combine it with artificial intelligence for medical decision-making.

## 2 Clinical applications of ultrasound in gynecological disease diagnosis

**Figure 1:**
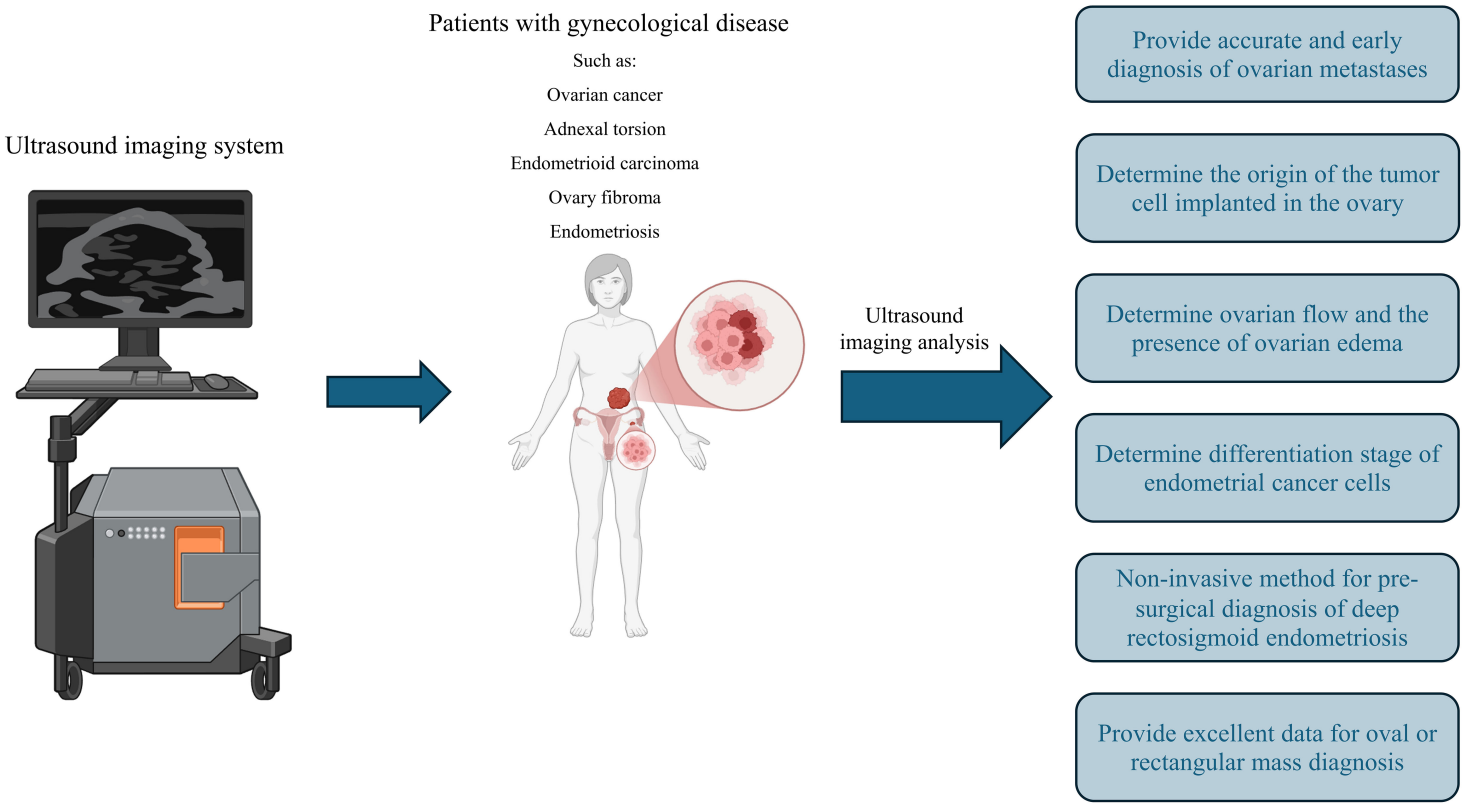
The application of ultrasound in gynecological disease. Ultrasound imaging can play a significant role in diagnosing numerous gynecological diseases, such as various cancers, endometriosis, and more. It can also assist in determining cancer stage and assessing ovarian reserve.

The US is a less invasive method with minimal side effects used to diagnose various diseases (16). If an experienced operator performs in the US, their results are precious in diagnosing diseases such as tumors, their size, metastasis, and follow-up treatment (17). In addition to the operator, the type of US device can also influence the diagnostic results. The conducted reviews should be recorded and used for further offline or artificial intelligence reviews. Another variable that can affect the rejection of US results is the patient (18). In some cases, obesity can create shadows that prevent correct diagnosis, and complementary methods must be used to confirm US results (**Table 1**) (19). In general, it is possible to use US in GyD such as fibroma and fibrosarcoma, adnexal torsion, acute abdomen, struma ovarii, ovarian dysgerminoma, decidualized endometriomas, extragastrointestinal stromal tumors, serous cystadenofibromas in adnexa, ovarian mature cystic teratomas, recurrent ovarian stromal cell tumors, ovarian yolk sac tumors, benign retroperitoneal pelvic peripheral-nerve-sheath tumors, etc (11, 20). The following section will explain this method for some of the mentioned diseases. **Figure 1** summarizes the characteristics of ultrasound in the diagnosis of gynecological diseases. (**Figure 1**).

**Table 1:** Summary of ultrasound application in observational and interventional clinical studies.

Study title	Study model	Enrollment	Status	Inclusion criteria	Year	NTC number
Microvascular Ultrasonographic Imaging for the Detection of Early Stage Epithelial Ovarian Carcinoma	Observational: Case-Only	100	Completed	1. Women must be greater than 21 years of age 2. Women must have a complex adnexal mass (as defined per ultrasound) which requires surgical intervention	2010	NCT00531570
Gynacological Imaging Reporting and Data System in Ovarian Masses by Ultrasonography	Observational: Other Time Perspective: Cross-Sectional	123	Completed	1. All women of different age groups diagnosed as having an ovarian mass. 2. Accidentally discovered ovarian mass in non-complaining female. 3. Patients known to have primary that may give metastasis to the ovaries.	2020	NCT03175991
Evaluating the Performance of Morphology Index in Surgical Decision-Making for Ovarian Tumors	Interventional: Single Group Assignment	179	Completed	1. 50 years of age or older 2. Documented ovarian abnormality on ultrasound 3. Undergone prior hysterectomy	2023	NCT02227654
Transvaginal Ultrasonography As a Screening Method for Ovarian Cancer	Interventional: Single Group Assignment	65000	Recruiting	1. 50 years of age or older 2. Documented family history of ovarian cancer 3. ECOG performance status of 0 to 2.34 4. Subjects having undergone prior hysterectomy	2024	NCT04473833
Contribution of Contrast Enhanced Ultrasound in the Diagnosis of Adnexal Torsion (AGATA)	Interventional: Single Group Assignment	11	Terminated	1. Woman over 18 years old 2. Planned surgical intervention for suspected adnexal torsion 3. Woman affiliated to a social security	2024	NCT04522219
Contribution of Contrast Enhanced Ultrasound for Diagnosis of Adnexal Torsion: a Randomized Controlled Trial (COVARIAN)	Interventional: Parallel Assignment	256	Not yet recruiting	1. Age 18 or over 2. Strong suspicion of adnexal torsion with surgery planned 3. No ongoing pregnancy or breastfeeding	2024	NCT06677554
Indirect Ultrasonographic Findings for Parametrial Involvement in Deep Endometriosis	Observational: Case-Control	1078	Completed	Patients with suspected deep endometriosis undergoing surgical approach	2022	NCT05239871
Efficacy of Double Contrast-enhanced Ultrasound of Pelvic in Preoperative Evaluation of Deep Endometriosis	Observational [Patient Registry]: Other	156	Recruiting	1. Sexual life history. 2. Surgery was performed within 2 months of the examination. 3. Subjects volunteered to participate in the study and signed the informed consent form.	2022	NCT05540821
Fusion Ultrasound for Diagnosis and Monitoring of Endometriosis Lesions (ENDOFUSION)	Observational: Case-Control	24	Active, not recruiting	1. Patients from 18 to 50 years old. 2. Patient with an indication for pelvic MRI and pelvic ultrasound.	2024	NCT04554602
Feasibility and Potential Aids of Intra-operative Endo-vaginal Ultrasound When Performing Rectal Shaving for Endometriosis (ECHOENDO)	Observational	10	Not yet recruiting	1. Age >18 years 2. Deep pelvic endometriosis with symptomatic rectal involvement 3. With surgical indication of rectal shaving validated in “RCP” or during the pre-operative consultation by the surgeon	2022	NCT05499884
Intraoperative Intra-abdominal Ultrasound for Endometriosis	Observational: Cohort	70	Recruiting	All the patients with suspicion of endometriosis who undergo surgery.	2023	NCT05812937
Prospective Validation and Comparison of Different Ultrasound Methods for Discrimination Between Benign and Malignant Ovarian/​Tubal Masses Prior to Surgery (IOTA7)	Observational: Cohort	1700	Unknown status	1. Woman presenting with an adnexal mass judged not to be physiological and likely to undergo surgery 2. Patients >18 years’ old 3. Patients that only underwent transabdominal scanning can be included in the study, but will be analysed separately.	2020	NCT02847832

### 2.1 Ovarian cancer and metastases

The results of transvaginal gray and color Doppler ultrasound examinations in patients with metastatic ovarian tumors originating from other tumors indicate that these tumors can be solid, multilocular, or solid-multilocular (21). The type of tumors that metastasize to the ovary can vary based on the origin of the tumor cells. Thus, tumors originating from the breast, uterus, stomach, or lymphoma are of solid type. This is while ovarian tumors originating from bile ducts, colon, appendix, or rectum are solid-multilocular or multilocular. Therefore, using ultrasound to check ovarian metastases can determine the origin of the tumor cells implanted in the ovary (22). Also, in another study, color Doppler was used to investigate the spread of a peripheral vessel called a lead vessel to the center of the mass formed in the ovary. The results of this study show that the lead vessel is observed in 35% of patients with solid mass and 52% of patients with solid-multilocular or multilocular tumors (23). Also, ultrasound has been used to compare primary ovarian tumors and tumors caused by metastasis from endometrial cancer. The results of this study show that the sonomorphological characteristics of these two types of tumors are different (24). Such diagnoses can help choose the type of treatment.

However, the use of molecular methods in combination with ultrasound can help in more accurate and early diagnosis of ovarian metastases. In a study conducted by F. Moro et al., CA125/CEA was combined with ultrasound to detect metastases of multilocular ovarian masses. After confirming ovarian tumors in patients, the levels of CA125, CEA, and the CA125/CEA ratio were evaluated. The results showed that, based on ultrasound data, CEA alone can be used for the differential diagnosis of ovarian metastases and ovarian neoplasms (25).

### 2.2 Adnexal torsion

This disease can occur due to the twisting of the adnexal vessels, which leads to irreparable damage to the ovarian tissue by cutting off the blood flow to the ovary (26, 27). Therefore, early diagnosis is essential in this disease. One of the primary methods for diagnosing this disease is through clinical and surgical examinations, which can be supplemented with ultrasound using Color Doppler (26, 28). The results of the retrospective studies conducted by F. Moro and colleagues investigated the torsion of the adnexal vessels in patients, which was confirmed using surgery. The results of this study indicate that the common symptoms can be diagnosed by ultrasound. This disease is characterized by the presence of free fluid in the pelvis, the whirlpool sign, enlarged adnexa, and ovarian stromal edema (OSE) (29). In another study, ultrasound was used to diagnose adnexal torsion with acute abdominal pain (30). The results of this study have shown that the sensitivity of ultrasound for diagnosing adnexal torsion is 84.4%, indicating a high diagnostic value (30, 31). Additionally, a 2023 meta-analysis study, which examined articles from 1990 to 2021, demonstrated that ultrasound examinations can be used to identify symptoms such as the reduction or absence of ovarian Doppler flow and the presence of ovarian edema. It was used to diagnose adnexal torsion with high sensitivity (32).

### 2.3 Endometrioid carcinoma

This cancer is the most common type of endometrial cancer in women and has recognizable molecular, macroscopic, and microscopic features (33). The presence of squamous differentiation, ciliated cells, and secretory cells usually recognizes this type of cancer (34). In addition, molecular features such as microsatellite instability (MSI), mutations in proto-oncogene genes like PTEN and k-RAS, and cell growth induction mutations such as FGFR2 can be used to identify endometrial carcinoma (35). The main treatment criterion for this disease is stage and histopathology. Surgery followed by radiotherapy can prevent the spread of tumor cells (36). It is imperative to diagnose this disease and prevent its progression. One of the primary methods for diagnosing this disease is biopsy, along with methods based on immunohistochemistry (33, 37). Thus, the observation of epithelial and mesenchymal malignant differentiation, as well as the immunohistochemical examination based on DNA mismatch repair (MMR) proteins, p53, and p16, can aid in diagnosing this disease (38, 39). However, performing a biopsy is invasive, and it seems that it can lead to the stimulation of tumor tissue and sometimes increase the metastasis of tumor cells. Therefore, the need for less invasive methods such as ultrasound is quite evident. In a retrospective study by F. Moro et al., ultrasound was used to diagnose endometrial carcinoma (20). This study evaluated the information of 239 patients examined by ultrasound. The results of this study show that endometrial carcinoma typically presents as large, multilocular-solid tumor masses (20).

Transvaginal ultrasound (TVS) or transrectal ultrasound (TRS) to check the differentiation stage of endometrial cancer cells also shows the good power of ultrasound in diagnosing two types of differentiation, well and moderate, and its results are consistent with the results of the biopsy of patients (40). In the case of endometrial carcinoma, it seems that one of the characteristics associated with high risk is myometrial infiltration. In the study conducted by L. Pineda, ultrasound was used to investigate this issue in hospitals (41). Also, the results have been compared with those obtained from the macroscopic mean. The extracted tissue was evaluated by pathologists who were unaware of the ultrasound results. The results showed that the use of TVS could be used as a method for assessing myometrial infiltration and as an alternative to intraoperative macroscopic examination, primarily when performed by an experienced examiner (41).

Additionally, a study by M. Cubo-Abert utilized TVS to investigate and compare its effectiveness with other methods, including magnetic resonance imaging (MRI), in the diagnosis of endometrial carcinoma. The results demonstrate that TVS is a reliable and suitable diagnostic method for low-grade endometrial carcinoma, and it can be used as the initial line of diagnosis (42). Therefore, it appears that using ultrasound to diagnose endometrial carcinoma can assist surgeons in making informed medical decisions and treating these patients.

### 2.4 Ovary fibroma and fibrothecoma

Fibroids and fibrothecoma are benign tumors of the ovary, which have a prevalence of about 4% among tumors related to the ovary (43). These diseases are typically associated with acid reflux and are referred to as Meigs syndrome. Considering the benign nature of these tumors, their early detection can help in the management of the disease (44). Fibroma and fibrothecoma can also be associated with Guerlain-Goltz syndrome, which affects the nature of the cells in the tumor mass and is usually related to the appearance of keratinocytes and basal cells (45). These diseases typically do not cause symptoms in patients (46). A study conducted by D. Paladini to diagnose fibroma and fibrothecoma using ultrasound reveals that approximately 50% of patients have ascites. The presence of fluid in the pouch of Douglas, the increase of CA125, and high color content in the diagnosis of this disease by ultrasound can lead to misdiagnosis of fibroid and fibrothecoma as malignancy (47). For this reason, it is said that the diagnostic features of these diseases using ultrasound are non-specific and need to be combined with other methods.

### 2.5 Endometriosis and adenomyosis

In recent years, TVS has been proposed as a primary test for pelvic ectopic endometriosis and endometriosis. In endometriosis, ectopic lesions form in different parts of the peritoneal cavity, ovaries, and other areas. Additionally, the presence of endometrial lesions in the myometrium is referred to as adenomyosis (48). Due to the effect of these diseases on the disruption of hormone levels and induction of infertility in patients, the use of alternative laparoscopic (ultrasound) methods, which are invasive, is essential. Also, in new studies, ultrasound has been used for high-precision diagnosis of deep ectopic lesions of the pelvis (49). Additionally, reviews and analyses conducted in a meta-analysis study have demonstrated that TVS, with or without the use of previous bowel preparation, is an accurate test for the non-invasive and pre-surgical diagnosis of deep rectosigmoid endometriosis (50, 51).

### 2.6 Tubal cancer

In a study, patients who were confirmed for tubal cancer by macroscopic and histopathological examinations were evaluated by color Doppler ultrasound and grayscale before surgery (52). The results of this study show that the images captured by ultrasound have three types of differential appearance in tubal cancer: a sausage-shaped cystic structure with solid tissue protruding into it like a papillary protrusion, a sausage-shaped cystic structure with a large solid component filling part of the cyst cavity and an excellent oval or rectangular mass. Therefore, tubal cancer can be diagnosed in patients by analyzing ultrasound images (53).

## 3 Artificial intelligence-assisted diagnosis

With the expansion and advancement of methods based on artificial intelligence (AI), these methods have found their way into medical fields (54, 55). In the past, algorithms and programming based on medical boards were used in diagnosis and treatment. However, conventional general programming algorithms generate outputs using given input data and rules, while artificial intelligence can create regulations and patterns using input and output data (56). Hence, AI can reliably predict outcomes from new input. It has been stated in many studies that with the specific development of artificial intelligence in a particular disease, it can be used in diagnosis, deciding on the type of treatment, and examining the treatment process (57). For example, in some studies, AI has been used to diagnose skin cancer and retinopathy in diabetic patients with high accuracy (58, 59). The use of AI in gynecology and obstetrics has had challenges (**Table 2**). However, recent advances have turned this method into a powerful tool, and using its potential can help improve the health of women and babies.

**Table 2:** The application of artificial intelligence in diagnosis and treatment of gynecological disease.

Study title	Study model	Enrollment	Status	Inclusion criteria	Year	NTC number
Predicting Outcome of Cytoreduction in Advanced Ovarian Cancer, Using a Machine Learning Algorithm and Patterns of Disease Distribution At Laparoscopy	Interventional	50	Not yet recruiting	1. Patients fit for cytoreductive surgery 2. Patients with a primary diagnosis of suspect Stage III-IV ovarian cancer 3. Patients selected for interval cytoreductive surgery after NACT	2024	NCT06017557
Predictors of Ovarian Cancer and Endometrial Cancer for Artificial-Intelligence-Based Screening Tools	Observational	2905	Recruiting	1. Women with gynecological symptoms 2. Women who underwent routine gynecological examination	2023	NCT05697601
Artificial Intelligence Model for Growth Prediction of Ovarian Cancer Organoids	Observational: Cohort	100	Recruiting	1. Patients received biopsy or puncture to obtain tumor tissues or malignant effusion 2. Patients must have histologically confirmed diagnosis of epithelial ovarian cancer	2024	NCT06317610
Artificial inTelligence in eNdometriosis-related ovArian Cancer and Precision Surgery in eNdometriosis-related ovArian Cancer	Observational: Case-Control	240	Unknown	1. Age>18 2. Suspected diagnosis of epithelial ovarian cancer 3. Radiological imaging available	2021	NCT05161949
An Artificial Intelligence Algorithm for Identifying Gynecologic Cancer Patients in Need of Outpatient Palliative Care	Interventional	400	Recruiting	Adult patient in Enhanced, Electronic health record (EHR)-facilitated Cancer Symptom Control (E2C2) with a diagnosis of advanced gynecologic malignancy	2024	NCT06182332

Artificial intelligence encompasses a set of digital computer systems equipped with features such as the ability to process information, exhibit intelligent behavior, demonstrate comparative performance, and engage in critical thinking, which enables the user to obtain a series of output data from the input data (60). In the case of medical issues, the input data are the results of patient examinations, and the output data can include test results, treatment decisions, tumor location, angiogenesis, fetal characteristics (complete or premature), and other cases. When discussing artificial intelligence, it is essential to understand that concepts such as machine learning (ML), deep learning (DL), and convolutional neural networks (CNN) overlap to a significant extent (61). However, there are also differences between them (62, 63). Considering that these concepts are used in the following text, their explanation can help to understand the content as well as possible. In applications based on machine learning, computer systems and software use input data to create a series of patterns.

Machine learning algorithms in AI can be divided into three categories: Unsupervised learning, supervised learning, and reinforcement learning (64, 65). The functions of these functions can be used in combination to diagnose and treat. During training, supervised learning algorithms learn from input data and labeled output targets to create a model that can classify or predict new data based on learned relationships. Usually, in this type of learning, some data whose results are known to the researchers are given to the used algorithm, and the information processed by it is compared with the real results that were already available to confirm the efficiency of the constructed algorithm (66). Unsupervised machine learning models differ from supervised models in that they learn and interpret relationships between key pieces of information in a dataset without predefined output data. They can reveal associations or clusters in data and complement supervised learning by identifying patterns that may not have been detected. This information can improve supervised algorithms and create new models (67).

Demographic and genetic factors significantly influence breast cancer risk. Studies emphasize age as a key criterion; middle-aged and older women exhibit higher incidence rates (68). Susceptibility varies by race, ethnicity, and geographic location, affected by genetic, environmental, and socioeconomic factors. Key risk factors necessitating tailored prevention plans include a family history of cancer and genetic mutations, such as those associated with BRCA1 and BRCA2 (69). Noteworthy economic disparities also impact access to care. Early menarche, late menopause, and hormonal treatments influence risk through hormonal exposure. The development of breast cancer also relies on metabolic factors such as insulin resistance and diabetes. Risk analysis is partly based on a patient’s medical history, including breast density and a history of previous malignancies.

Artificial intelligence enhances detection, diagnosis, treatment, risk assessment, and prevention, particularly through improved mammography (70). While there are concerns about false positives, it aids radiologists in identifying lesions. Despite challenges such as data privacy and ethical concerns, artificial intelligence also aids in radiotherapy planning and genetic risk analysis (71).

Although there are few investigations, AI in mammography screening looks to have some potential (72). In women 50–69 at 12 locations in Germany, the PRAIM study matched AI-supported double reading against conventional double reading (73). Between July 2021 and February 2023, 463,094 women were screened, and 260,739 of them received AI assistance. At 6.7 per 1,000, the artificial intelligence team had a breast cancer detection rate that was 17.6% higher than the control group (73). The recall rate of the AI group was lower than that of the control, and the positive predictive values for biopsies and recalls were also higher. Artificial intelligence could raise mammography screening indicators (74).

## 4 Ultrasound and artificial intelligence in gynecological disease

One of the primary methods used in gynecology and obstetrics is the combination of ultrasound and AI (75). The retrospective analysis of ultrasound images from patients, combined with the creation of a database that can be integrated with AI, can facilitate a more accurate diagnosis of women’s diseases by comparing new ultrasound images. This type of method also leads to a reduction in the time from imaging to diagnosis (76). Additionally, the use of this technology can lead to a reduction in human error in the diagnosis of GyD. Therefore, by utilizing various types of artificial intelligence tools, ultrasound images can serve as input data to create an algorithm (77). The combined use of different AI tools will significantly help obstetrics and gynecology in the future. In the following, we will discuss some artificial hash tools that GyD can use (**Table 3**).

**Table 3:** Application of ultrasound and artificial intelligence in combination for diagnosis and treatment of gynecological disease.

Study title	Study model	Enrollment	Status	Inclusion criteria	Year	NTC number
Detection of Ovarian Cancer Using an Artificial Intelligence Enabled Transvaginal Ultrasound Imaging Algorithm	Interventional: Parallel Assignment	10000	Unknown status	1. Women scheduled for Transvaginal Ultrasound examination for adnexal lesions. 2. Age >18 years	2021	NCT04214782
Research and Application of Ultrasonic Intelligent Diagnosis System for Ovarian Mass	Observational: Other	100000	Not yet recruiting	1. During gynecological ultrasound examination, at least one patient with persistent ovarian tumor was found. 2. The patient underwent surgical treatment and the histopathological results.	2024	NCT06528236
Delivery Outcomes by AIDA (ARTIFICIAL INTELLIGENCE DYSTOCIA ALGORITHM) Analysis (AIDA)	Observational [Patient Registry]	1000	Recruiting	All patients in pregnancy, nulliparae, candidates for spontaneous or induced labor, monitored by intrapartum ultrasound, collecting the ultrasound parameters of the labor progress. 1. Pregnants in labor, at first pregnancy 2. Gestational age ≥37 weeks of gestation	2024	NCT06664112

**Figure 2:**
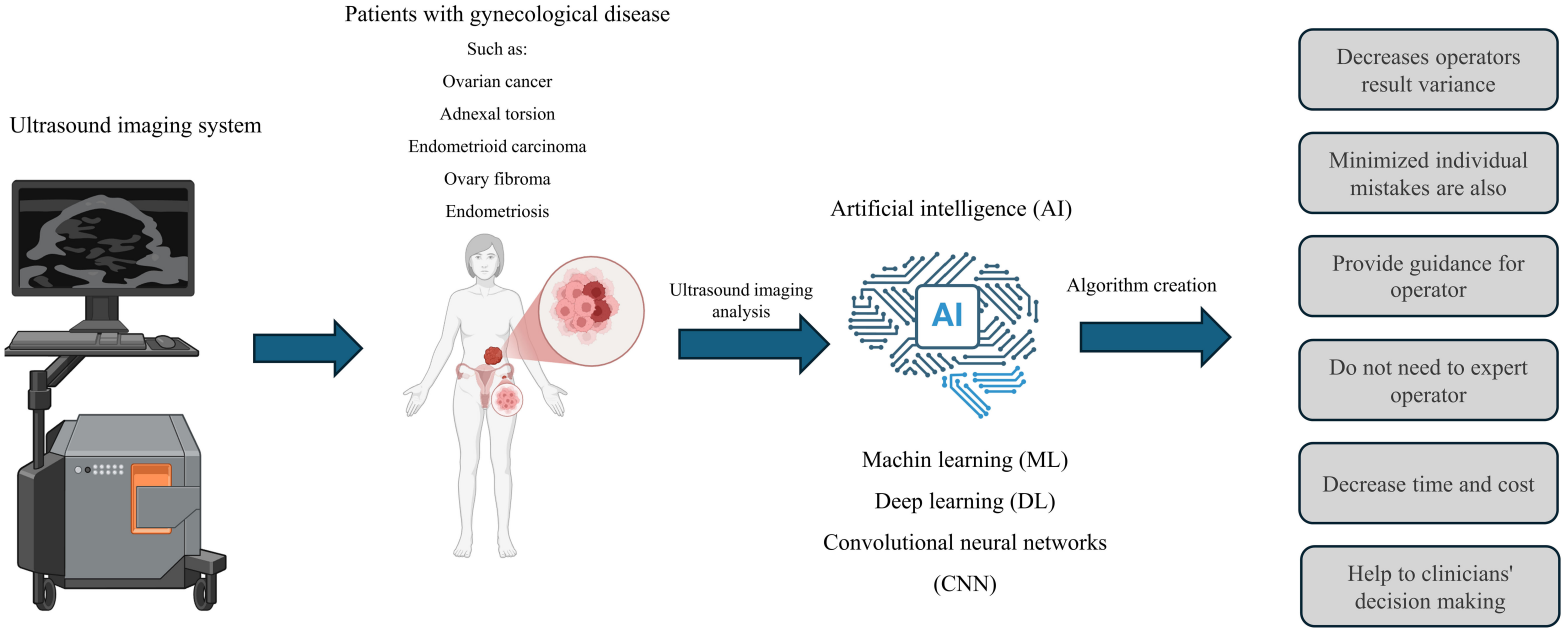
The application of ultrasound and its integration with artificial intelligence in gynecological disease. The use of artificial intelligence can lead to the development of algorithms that assist in diagnosing women’s diseases. This combination can enhance diagnostic accuracy, decrease the reliance on professionals, speed up the diagnostic process, and lower costs. However, artificial intelligence cannot replace experts.

Probe guidance, which is also called AI-GUIDE, is one of the most widely used AI tools, which is used in echocardiography and in working with probes that are necessary for taking pictures (78). Using a Probe guidance application, which guides the operator in orientation and how to manipulate the ultrasound probes to obtain a suitable biometric screen for photographing the fetus, is beneficial. Therefore, this application can help teach ultrasound and entrust the initial scans to general doctors who do not perform this test (**Figure 2**). Thus, the need for the permanent presence of experts is reduced, and errors caused by individual mistakes are also minimized (79). In the study conducted by Richard Droste et al. on Probe guidance and using probe movement data obtained from 464 tests performed by expert operators, it was shown that this application, by combining the motion signal of the Inertial Measurement Unit (IMU) and creating the US-GuideNet network, helps guide the operator (80).

In another study, two common standard methods were employed to examine the participants and their fetal anomalies: one used by a professional operator and the other with the assistance of artificial intelligence. The results show that using Intelligent Fetal Imaging and Diagnosis (iFIND) to separate freehand scanning from image capture and measurement leads to faster scanning and improved workflow. It appears that AI can automate repetitive tasks, potentially leading to increased attention being paid to identifying fetal abnormalities (81). The study conducted by L. Drukker and colleagues also used deep learning algorithms to track the movement of the operator’s eyes and create an algorithm for performing ultrasound examinations. They also used the resulting data to develop an algorithm. The results of this study indicate that the operator’s influence can impact the outcome, and artificial intelligence can help mitigate this variation (82). Another application of artificial intelligence is anomaly highlighting, which can identify and report unusual findings about the fetus during ultrasound scans in standard planes and aid in sonographic referrals (83).

Another artificial intelligence tool that has many users is Cyst classification. The cysts in the ovaries in polycystic ovary syndrome (PCOS) and cancers can affect ovarian functions in fertility as well as women’s health (84). Various factors such as genetics, nutrition, activity level, and smoking can have a significant effect on the onset and spread of this disease (85). Classification of cysts is essential because they require different treatments. One of the primary methods of classifying cysts in PCOS and various types of ovarian cancers is the method described by the International Ovarian Tumor Analysis (IOTA) (86). The diagnosis is based on IOTA ultrasound criteria. According to IOTA, ovarian cysts are divided into benign and malignant categories, depending on their characteristics (87). The ovarian cysts are divided into two categories: complex and simple (88). However, intermediate states have also been defined. In addition, the AROMA study classified ovarian masses into three groups: solid, cystic, and motley, and the diagnostic accuracies were separately compared to other studies that classified ovarian masses into benign and malignant (89).

In a retrospective study using archived data of IOTA-related criteria for the diagnosis of fetal ovarian cysts in 51 patients (90). With the advancement of AI-based methods, giving IOTA-related criteria to artificial intelligence tools can be used for cyst classification. The use of these applications can lead to increasing the decision-making speed of doctors, reducing human errors in diagnosis, and helping to choose the proper treatment (90).

One of the primary diseases that can be evaluated, diagnosed, treated, and followed up by the combination of ultrasound and AI is ovarian and uterine cancers (91). A meta-analysis study published by Sian Mitchell et al. reports that, as of October 2022, 14 articles have utilized AI to analyze data from ultrasound scans, investigating anomalies and ovarian cancer in patients. The statistical analyses of these studies show that AI with high overall sensitivity (81%) and high specificity (92%) can diagnose these cases in patients (89). In a systematic review study published by F. Moro et al. in 2024, they reviewed studies that utilized ultrasound and artificial intelligence in gynecologic oncology. After selecting articles based on existing criteria and using RAYYAN QCRI and QUADAS-AI software, 50 articles were extracted, and their results were reported. A notable point is that most studies were conducted in the field of ovarian cancer (37 out of 50). In most of these studies, machine learning-based methods have been used to detect ovarian masses. Therefore, this study has introduced artificial intelligence in combination with ultrasound as a powerful and efficient tool for diagnosing ovarian tomographic masses (92). Additionally, Francesca Arezzo and colleagues stated that, in addition to the role that AI combined with ultrasound can play in diagnosing tumor masses, it can also predict progression-free survival (PFS) in ovarian cancer patients. For this purpose, researchers retrospectively combined data from ultrasound (2019 and 2018) with machine learning-based modeling to predict 12-month progression-free survival (PFS). The data analysis results show that using the Random Forest algorithm (RFF) with 90% accuracy and 90% recall can accurately determine the 12-month progression-free survival (PFS) of patients with epithelial ovarian cancer (93). Between 2009 and 2023, the research group of Amor and colleagues conducted approximately 11 studies evaluating the performance of artificial intelligence in classifying and analyzing data from ultrasound (Gynecologic Imaging Reporting and Data System (GI-RADS)) in adnexal masses (94).

## 5 Medical decision-making using ultrasound and artificial intelligence

Artificial intelligence has been a new and transformative tool since its inception. From its primary conditions to today’s advanced states, it has been in medicine and related services (95, 96). One field in which artificial intelligence for making treatment decisions has been tested is diseases related to gynecology and obstetrics (97). As mentioned in the previous section, the use of artificial intelligence tools enhances the speed and accuracy of disease diagnosis. Also, this tool can help in treatment after diagnosis (98). In such a way, by entering information related to medical history, lifestyle, and genetics, it is possible to personalize disease-based decisions in people (99). However, issues such as privacy may be questioned in these approaches, and informed consent must be obtained from patients to use the information and enter it into a database (100). Algorithms related to decision-making in artificial intelligence should also be well-evaluated so as not to make mistakes (77). One of the areas where artificial intelligence can be used in the treatment of GyD is its help in surgeries.

Artificial intelligence can be used in various stages of surgery, including 1) preoperative planning and 2) real-time guidance during procedures (54, 101). One of the primary pieces of information used in artificial intelligence for surgical applications is images obtained through ultrasound. By analyzing these images with artificial intelligence tools, the location of masses and vessels is determined to perform more accurate surgeries and prevent operator errors (102).

In addition, artificial intelligence can be integrated into robotic surgery through advanced algorithmic programming, allowing surgical interventions and treatments to be performed entirely by the robot and transferring all the information from the surgery to the specialist in real-time (103, 104). Therefore, in general, artificial intelligence and machine learning have the potential to significantly improve the field of GyD-related surgeries by reducing risk, increasing accuracy, reducing complications, and improving patient outcomes (77). With the continued development of this technology, in the not-too-distant future, we will witness an increasing number of surgical systems and applications that utilize artificial intelligence in clinical practice, including tumor removal surgeries, the removal of lesions related to endometriosis, and fully automated laparoscopy by a robotic surgeon (105).

## 6 Limitations and future directions

Artificial Intelligence cannot replace doctors; it can merely assist in clinical practice by supporting doctors’ decisions and preventing other errors. The performance of AI algorithms depends on the availability of high-quality and representative datasets. In oncogynecology, AI holds promise for early diagnosis and improved patient outcomes. Such problems include but are not limited to the following: much data is needed, biased data results in biased AI models, and limited interpretability exists in the AI model, besides the fact that it is hard to handle uncertainty (106). AI raises ethical challenges in the diagnosis of GyD due to concerns over data privacy, potential algorithmic bias, transparency, and liability. Confidentiality and protection of patient data are paramount, as AI models trained on sensitive data may prove vulnerable to data breaches. Algorithmic bias arising from biased training data will lead to biased predictions and exacerbate health inequities (107).

Furthermore, there is an ethical concern about discrimination and the replacement of human doctors by AI systems (108). In other specialized areas, such as gynecology and obstetrics, where data is relatively scarce, the development of highly accurate AI models becomes limited. Any biased data used for training AI models may result in less reliable predictions for specific patient groups (109). AI models are large “black boxes,” and it is challenging even for doctors to comprehend their predictive results, let alone be confident in them (97, 110).

The clinical use of AI presents several advantages, but also a long list of challenges and unknowns. One significant issue will likely be the impact of AI on jobs, rather than the creation of unemployment. Automation, including AI, will enhance efficiency and job satisfaction for professionals, such as sonographers, by automating routine scanning tasks, thereby allowing them to devote more time to patient care (110, 111). Machines offer a consistency that human clinicians may lack for various reasons. Early applications of AI are likely to involve repetitive tasks in imaging, addressing the shortage of imaging experts, and meeting the surging demand for diagnostic imaging. Concerns are also raised regarding the generalization of AI into clinical diagnosis and management, as AI models often depend entirely on imaging data without considering vital clinical context, such as age or familial risk (110).

Other issues are related to the safety of adaptive AI systems, as existing regulations prescribe static models that do not change over time. AI’s “black box” nature is unnerving to clinicians who want to understand possible biases in AI models, as human input can also bias it (112). Moreover, resource-intensive processes for annotating data result in prejudice and need strategies such as using pre-trained models or smaller model sizes for deployability (113, 114). While high expectations for AI exist, convincing clinical studies have yet to be widely reported. The last one deals with professional liability in the light of AI-assisted incorrect diagnoses, which has raised heated and ongoing debates among regulators, lawyers, and clinicians (115, 116).

## 7 Conclusion and future perspective

In recent years, AI technologies in gynecologic surgery have revolutionized the way surgeries are performed. Artificial intelligence in this field should no longer be viewed as a dream of the future but as a reality shaping the course of planning, conducting, and evaluating gynecologic surgery (114). Robotics, such as the da Vinci Surgical System (117), has been used in increasingly complex procedures, like hysterectomies and myomectomies, with even greater precision and control (118, 119). It also involves preoperative planning and decision-making, utilizing machine learning and predictive analytics to analyze imaging data and develop personalized surgical plans that take into account each patient’s unique anatomy and medical conditions. Imaging and diagnostics utilize AI to enhance the diagnostic accuracy of gynecologic conditions, including ovarian cysts, fibroids, and cancers. With the power of AI, minute information from imaging data that might have escaped the human naked eye can be analyzed, resulting in the early diagnosis of diseases.

AI and human experts each offer unique skills in the field of gynecological illnesses. In particular, AI excels at analyzing large datasets to identify trends and aid in the early detection of disorders like cervical and ovarian cancer. Conversely, humans provide essential elements such as clinical judgment, nuanced interpretation, and patient-centered care. AI-assisted tools can enhance human capabilities, potentially resulting in faster and more accurate diagnoses, improved treatment strategies, and ultimately better patient outcomes.

AI also enhances postoperative care by monitoring recovery, predicting complications, and providing personalized recommendations. Challenges, however, include ethical issues, data privacy concerns, misinterpretation of clinical scenarios, high costs, and the need for specialized training among healthcare professionals. Notwithstanding, AI technologies are shaping gynecological surgery with precision, efficiency, and personalization; hence, they promise to improve surgical outcomes and patient care in gynecology. These ever-evolving AI technologies will further revolutionize this most essential field of medicine. The integration of AI in gynecological surgery shows great potential for innovation and improvement in patient care.

AI technologies, such as machine learning and robotics, can enhance personalized medicine by analyzing patient data in real-time and offering customized surgical plans. AI-driven virtual reality simulations can provide surgeons with realistic training experiences, while AI-enhanced surgical tools promise enhanced precision and flexibility during procedures. However, challenges such as regulating AI integration for patient safety and data privacy must be addressed through collaboration among the medical community, regulators, and ethicists. The future of AI in gynecological surgeries appears promising. Still, it is crucial to strike a balance between technological advancements and ethical considerations, as well as patient-centric care, through ongoing collaboration among gynecologists, AI researchers, and policymakers.
